# Multidirectional Spin–Orbit Torque Magnetization Dynamics in beyond Room Temperature Van der Waals Magnet Devices

**DOI:** 10.1021/acs.nanolett.6c02494

**Published:** 2026-07-14

**Authors:** Bing Zhao, Lakhan Bainsla, Soheil Ershadrad, Prabhav N. Sumant, Roselle Ngaloy, Johanna Rosen, Biplab Sanyal, Johan Åkerman, Saroj P. Dash

**Affiliations:** ‡ Department of Microtechnology and Nanoscience, 11248 Chalmers University of Technology, SE-41296 Göteborg, Sweden; § Department of Physics, Indian Institute of Technology Ropar, Roopnagar 140001, India; ⊥ Department of Physics, Thin Film Physics, Chemistry and Biology (IFM), 4566 Linköping University, Linköping SE-58183, Sweden; ∥ Wallenberg Initiative Materials Science for Sustainability (WISE), Department of Physics, Chemistry and Biology (IFM), Linköping University, Linköping 58183, Sweden; ¶ Department of Physics and Astronomy, 8097 Uppsala University, Box 516, Uppsala SE-75120, Sweden; # Department of Physics, 3570 University of Gothenburg, SE-41296 Göteborg, Sweden; @ Center for Science and Innovation in Spintronics, Tohoku University, 2-1-1 Katahira, Aoba-ku, Sendai 980-8577 Japan; ∇ Research Institute of Electrical Communication, Tohoku University, 2-1-1 Katahira, Aoba-ku, Sendai 980-8577, Japan; ○ Wallenberg Initiative Materials Science for Sustainability, Chalmers University of Technology, SE-41296 Göteborg, Sweden; $ Graphene Center, Chalmers University of Technology, SE-41296 Göteborg, Sweden

**Keywords:** spin−orbit torque (SOT), vdW magnets, 2D materials, room temperature, ST-FMR, second harmonic Hall, magnetization dynamics

## Abstract

van der Waals (vdW) magnets with room-temperature ferromagnetism offer exciting opportunities for energy-efficient spintronic devices, yet their magnetization dynamics remain largely unexplored despite their importance for high-speed memory technologies. Here, we investigate spin–orbit torque phenomena in the room-temperature vdW magnet (Co_0.15_Fe_0.85_)_5_GeTe_2_ (CFGT)/Pt heterostructure using spin-torque ferromagnetic resonance and second-harmonic Hall measurements. Alongside a conventional in-plane spin Hall conductivity of 3.68 × 10^5^ (ℏ/2e) (Ω m)^−1^, we identify a sizable out-of-plane component of −0.33 × 10^5^ (ℏ/2e) (Ω m)^−1^ that generates unconventional damping-like torques. Density functional theory and Monte Carlo simulations suggest that this torque can originate from interface-induced spin reorientation arising from a modified magnetic anisotropy landscape and strongly enhanced Dzyaloshinskii–Moriya interaction at the CFGT/Pt interface. The combination of low effective magnetization (0.321 T), moderate Gilbert damping (0.027), and efficient multidirectional torques highlights vdW magnets as promising platforms for next-generation spintronic devices.

## Graphical abstract

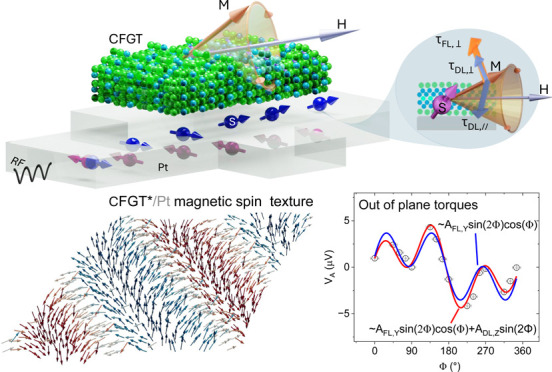

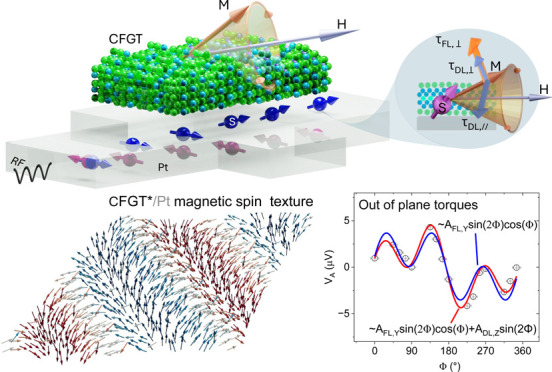

Continued technological advancements drive the need for smaller, faster, nonvolatile, and energy-efficient spintronic devices for information and communication technologies.^1,2^ Spintronic memories based on spin-transfer torque (STT) phenomena have already been integrated into microcontrollers for autonomous systems, automobiles, space, gaming, artificial intelligence, and machine learning platforms.^3–5^ In recent years, the chip industry has become interested in spin–orbit torque (SOT) nonvolatile memory for high-performance computing, due to its energy efficiency, faster speeds, and higher endurance and retention compared to its counterparts.^6–11^ However, key challenges remain in achieving lower energy consumption, magnetic field-free operation, and tunability, necessary for widespread adoption in higher-level memory, memory-in-logic, and applications in artificial intelligence and neuromorphic computing.^4–6,12,13^

The emergence of two-dimensional (2D) vdW magnets has opened a new paradigm for spintronic device engineering because of ultralow dimensionality, tunable magnetic properties, and the ability to enable energy-efficient and field-free magnetization switching mechanisms.^14–18^ Recent demonstrations of room-temperature vdW ferromagnets offer robust magnetization and a high degree of anisotropy control,^17,19^ spanning both ferromagnetic and antiferromagnetic regimes,^17,19–23^ as well as exotic spin textures^24,100^ and coexisting magnetic orders.^25^ These properties have enabled proof-of-concept magnetic tunnel junctions,^26–28^ lateral spin-valves^22,23,100^ and spin–orbit torque (SOT) memory devices.^25,29–34^ However, systematic experimental studies of magnetization dynamics, specifically their spin–orbit torque efficiencies, magnetization damping, and charge-to-spin conversion, remain sparse but are essential for realizing fast and energy-efficient spintronic technologies.

Here, we report multidirectional SOT magnetization dynamics using the above room temperature vdW magnet (Co_0.15_Fe_0.85_)_5_GeTe_2_ (CFGT) in heterostructure with Pt. Using both spin-torque ferromagnetic resonance (ST-FMR) in the frequency range of 2–14 GHz and second (2^nd^) harmonic Hall measurement techniques, we observe a large in-plane spin Hall conductivity and a significant unconventional out-of-plane spin Hall conductivity. Further DFT calculations combined with Monte Carlo simulation show that proximity with Pt can introduce complex noncolinear spin textures in the CFGT, which can be attributed to the strongly enhanced Dzyaloshinsky–Moriya interaction and weakened magnetic anisotropy energy with tilted magnetic easy axis. These results reveal crucial parameters governing efficient spin dynamics and unconventional SOT in vdW magnets for next-generation spintronic devices.

We were motivated to study magnetization dynamics in vdW magnet CFGT because of their above-room-temperature ferromagnetism with strong in-plane magnetic anisotropy.^22^ Here, we employed the spin–orbit torque (SOT) induced ST-FMR and 2^nd^ harmonic Hall measurements to investigate the magnetization dynamics of the vdW magnet CFGT/Pt heterostructure (Figure 1a). For the ST-FMR device fabrication, the CFGT crystals were first exfoliated onto a Si/SiO_2_ substrate inside a glovebox, followed by Pt deposition to create the CFGT/Pt heterostructure (see the Methods section). Subsequently, the CFGT/Pt heterostructure was patterned into rectangular microbar devices with an integrated microwave guide with Ti/Au contacts for the ST-FMR measurements (Figure 1b). The acquired measurements unveiled information about effective magnetization μ_0_ *M*_eff_, effective Gilbert damping constant α, magnetic anisotropy *H*_k_, and SOT efficiency at room temperature.

**Figure 1:**
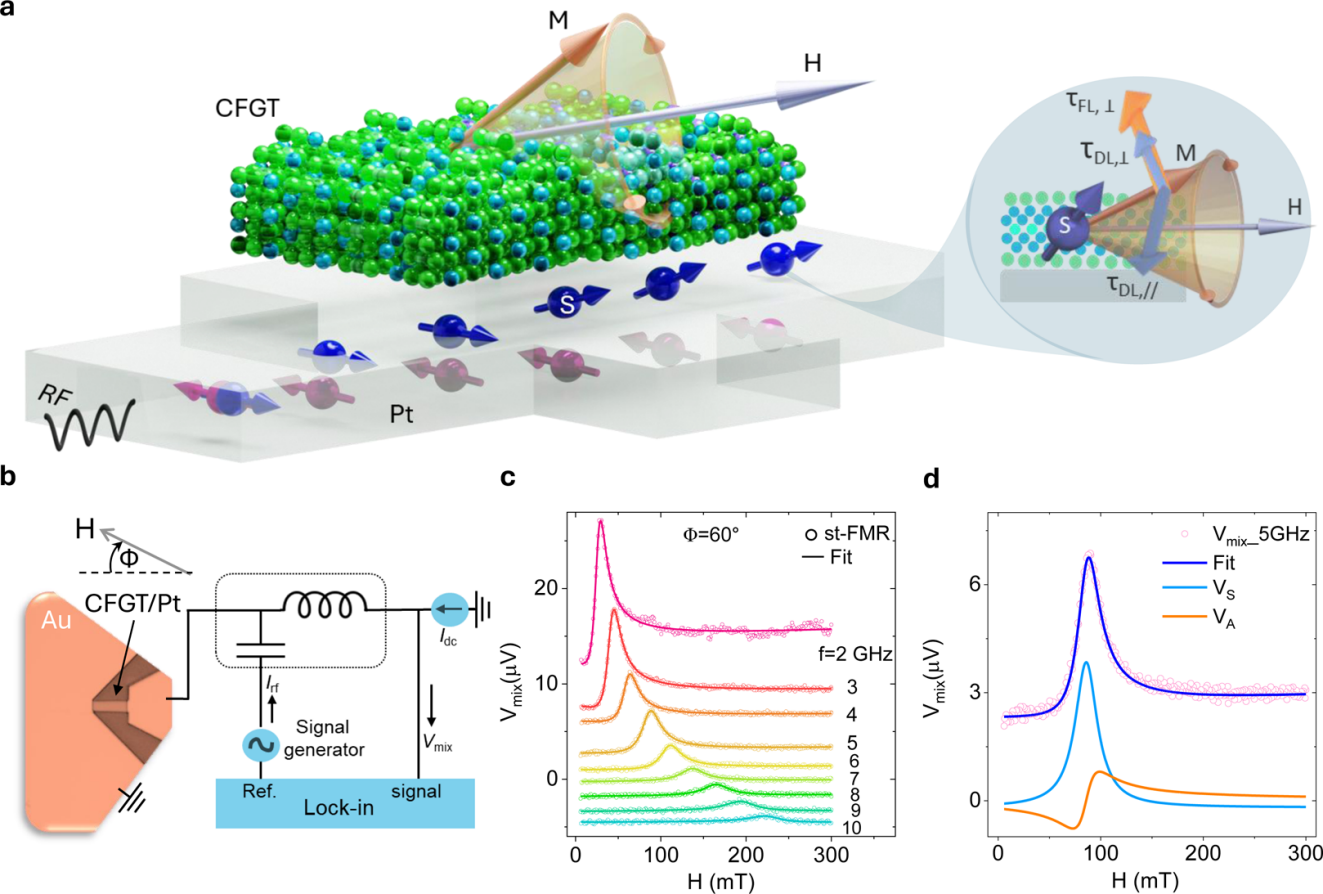
Magnetization dynamics measurements using CFGT/Pt heterostructure at room temperature. (a) Schematics of the CFGT/Pt heterostructure with magnetization M oscillation driven by SOTs and external B field. The inset shows the multidirectional spin current induced by conventional and unconventional SOT in the CFGT/Pt heterostructure. (b) Schematics of the spin-torque ferromagnetic resonance (ST-FMR) measurement setup with CFGT/Pt heterostructure device. (c) Representative ST-FMR data, showing *V*_mix_ as a function of the magnetic field at different radio frequencies in the range of *f* = 2–10 GHz at Φ = 60°. (d) ST-FMR signal at 5 GHz with the fitted symmetric (V_S_) and antisymmetric (V_A_) components. Measurements are performed on the CFGT­(25 nm)/Pt­(10 nm) ST-FMR device.

In ST-FMR measurements, an in-plane radio-frequency (RF) current *I*_RF_ is applied along the CFGT/Pt stripe, while an in-plane magnetic field *H* is applied at an angle Φ with respect to the *I*_RF_. The spin–orbit coupling in Pt generates a spin current perpendicular to the direction of *I*_RF_, which is injected into the adjacent CFGT layer and exerts a torque on the magnetization of CFGT, causing it to precess. The *I*_RF_ causes the anisotropic magnetoresistance (AMR) to oscillate and produces a DC mixing voltage *V*_mix_, which is then measured using a lock-in amplifier. The representative ST-FMR signals *V*_mix_ for the CFGT/Pt device at room temperature are shown in Figure 1c. The obtained *V*_mix_ is fitted by eq 1,^35–37^

1 $$V_{\mathrm{mix}}=V_{S}F_{S}\left(H\right)+V_{A}F_{A}\left(H\right)$$

where $F_{S}\left(H\right)=\frac{\Delta H^{2}}{\Delta H^{2}+{\left(H-H_{R}\right)}^{2}}$ and $F_{A}\left(H\right)=F_{S}\left(H\right)\left[\frac{\left(H-H_{R}\right)}{\Delta H}\right]$ are symmetric and antisymmetric Lorentzian functions, respectively. *H*, Δ*H*, and *H*_R_ are the applied external magnetic field, ferromagnetic resonance line width, and ferromagnetic resonance field, respectively. *V*_S_ and *V*_A_ are the amplitudes of symmetric and antisymmetric signals and are proportional to the current-induced in-plane torque and out-of-plane torque, respectively.^38^ The obtained *V*_mix_ at 5 GHz is shown in Figure 1d, where symmetric and antisymmetric components are extracted and shown for reference. The extracted values of Δ*H* and *H*_R_ are used to obtain the effective magnetization μ_0_ *M*_eff_, by fitting the frequency *f* versus *H*_R_ data to the Kittel equation^37,39,40^ $f=\left(\frac{\gamma }{2\pi }\right)\mu_{0}\sqrt{\left(H_{R}-H_{k}\right)\left(H_{R}-H_{k}+M_{eff}\right)}$ as given in Figure 2a. The Gilbert damping constant α is evaluated by fitting the Δ*H* versus *f* using $\Delta H=\Delta H_{0}+\frac{2\pi \alpha f}{\gamma }$ , as shown in Figure 2b. The μ_0_ *M*_eff_ = 0.321 ± 0.014 T and α = 0.027 ± 0.001 are obtained for the CFGT/Pt device, as given in parts a and b of Figure 2, respectively (additional results at Φ = 240° are presented in Supplementary Figure S1).

**Figure 2:**
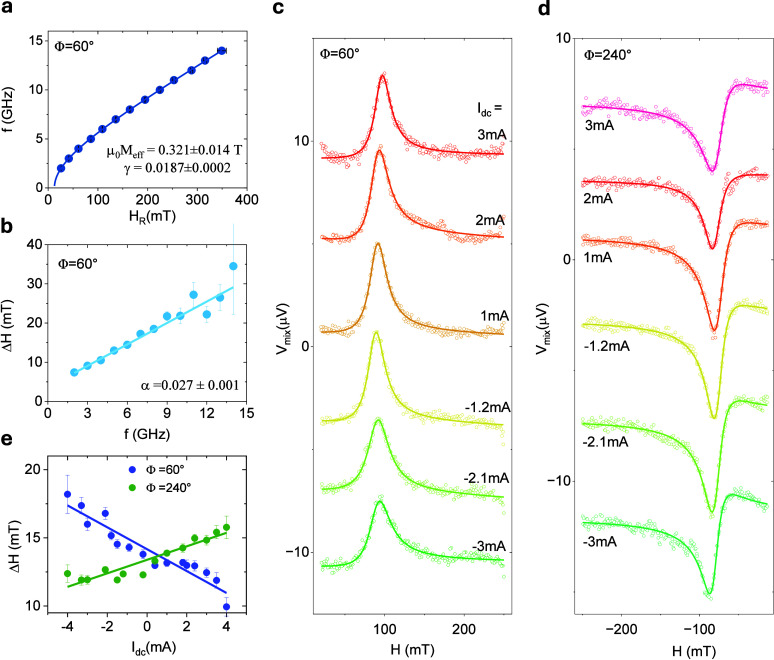
Evaluation of magnetization dynamic parameters and effective spin–orbit torque efficiency from DC bias-dependent ST-FMR measurements. (a) Fitting of resonant frequency *f* versus the extracted resonant field *H*_R_. (b) Fitting of resonant extracted line width Δ*H* versus resonant frequency *f* (GHz). The error bars are calculated from the standard deviation of the signal background. (c and d) DC current tuned ST-FMR plots for positive/negative fields at a frequency of 5 GHz using different values of *I*_dc_. Hollow dots denote experimental data while solid lines correspond to fitting performed using eq 1. (e) Line width versus DC current plot at 5 GHz for positive (blue line) and negative (dark green line) fields. The fitted slope of these lines is used to calculate the SOT efficiency of damping-like torque using eq 2. All the solid curves are the fitting results.

To estimate the effective damping-like SOT efficiency, the DC bias-dependent ST-FMR measurements are done on the ST-FMR device as well, and the representative data at different values of DC current (*I*_dc_) are shown in Figure 2c,d. The resonance line width, Δ*H* at different *I*_dc_ is extracted, and shown in Figure 2e. The strength of damping-like SOT efficiency is estimated using the slope of linearly fitted Δ*H* versus *I*_dc_ data,^35–37^

2 $$\xi_{\mathrm{DL}}^{\mathrm{eff}}=\frac{2e}{\hbar }\frac{\left(H_{R}+0.5M_{\mathrm{eff}}\right)\mu_{0}M_{S}t_{\mathrm{CFGT}}}{\sin\;\phi }\frac{\gamma }{2\pi f}\frac{\delta \Delta H}{\delta \left(I_{\mathrm{dc},\mathrm{Pt}}\right)}A_{C}$$

where Φ is the azimuthal angle between *I*_dc_ and *H*. *M*_eff_, μ_0_ *M*_S_, *t*_CFGT_, and $\frac{\gamma }{2\pi }$ are the effective magnetization, saturation magnetization, thickness, and effective gyromagnetic ratio of the CFGT layer, respectively. *e* and ℏ are the elementary charge and the reduced Planck’s constant. *I*_dc,Pt_ is the current in the Pt layer, and *A*_C_ is the cross-sectional area of the ST-FMR microbars. The effective spin Hall conductivity (σ_SHC_ = σ_c_ξ_DL_^eff^) values are evaluated to be 1.13 × 10^6^ (ℏ/2e) (Ω m)^−1^ for CFGT/Pt heterostructure. Noticeably, the effective SOT efficiency value obtained from DC bias-dependent measurements might have unwanted contributions due to thermal effects, which could result in deviations in SOT efficiency compared to its intrinsic values.^41^

To gain more insight into different SOT components, we also performed the in-plane angle dependence of the ST-FMR measurements, as shown in Figure 3a,b. The symmetric (*V*_S_) and antisymmetric (*V*_A_) components are extracted by fitting the ST-FMR signals at different in-plane angles Φ with eq 1, as illustrated in Figure 3c,d. Traditionally, the relation of the symmetric and antisymmetric components as a function of the in-plane angle Φ can be expressed as follows:^38,42^

3 $$V_{S}=S_{\mathrm{DL},Y}\;\sin\left(2\Phi \right)\;\cos\left(\Phi \right)$$

4 $$V_{A}=A_{\mathrm{FL},Y}\;\sin\left(2\Phi \right)\;\cos\left(\Phi \right)$$

**Figure 3:**
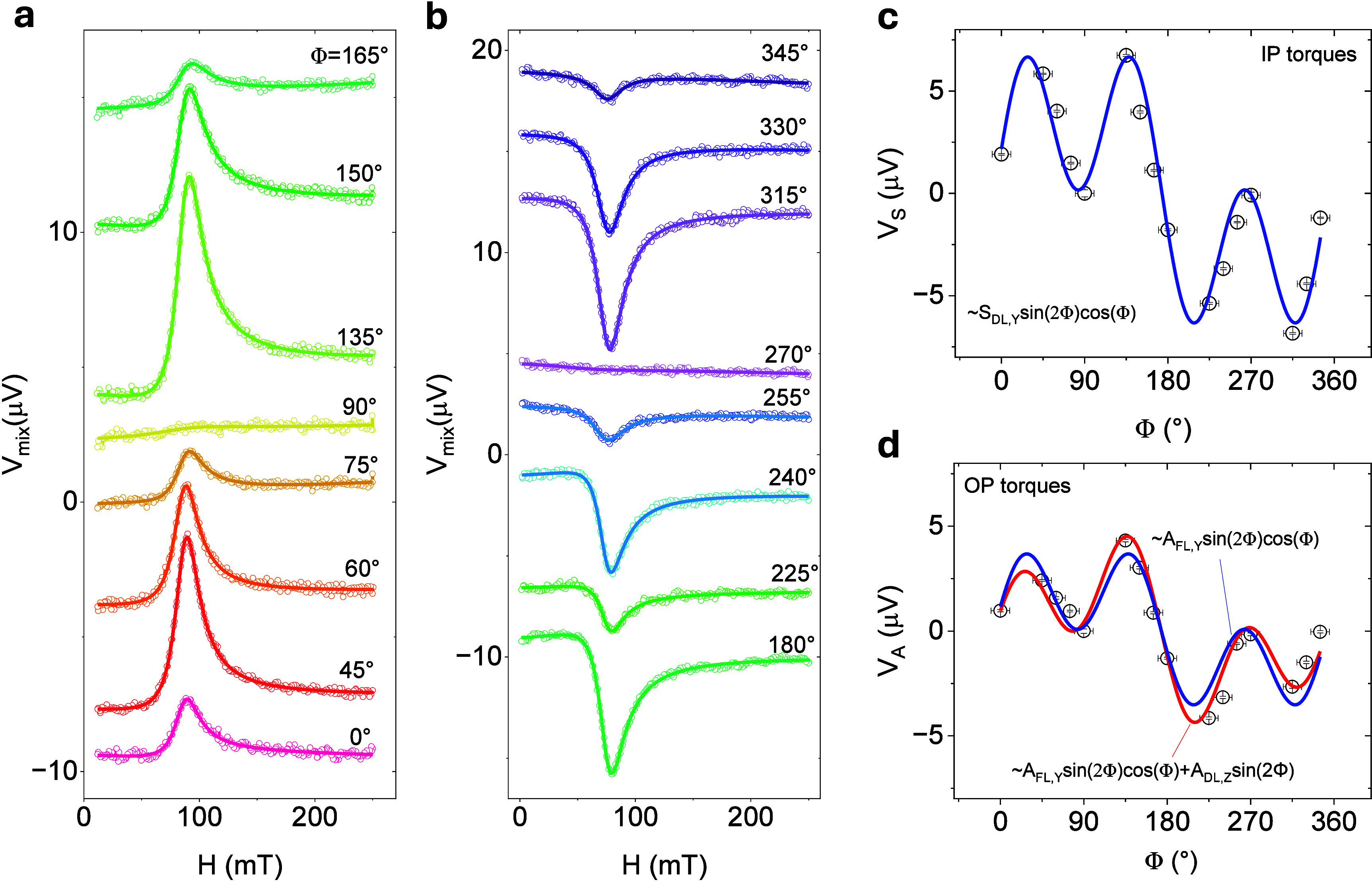
Unconventional spin–orbit torque in CFGT/Pt heterostructures from angular ST-FMR measurements. (a and b) Angular dependence of the ST-FMR signals at a frequency of 5 GHz. The open circles denote the output *V*_mix_ signal, and the solid lines represent the fitting by eq 1. (c and d) Angular dependence of the extracted symmetric component *V*_S_ and antisymmetric component *V*_A_, respectively. Solid curves denote the fitting to the data using equations indicated in the figures.

*S*_DL,*Y*_ and *A*_FL,*Y*_ are the coefficients for the damping and field-like torques with spin polarizations along the *y* direction. However, we found that the antisymmetric component is not fitted properly with eq 4 (blue curve in Figure 3d). Then another term *A*_DL,*Z*_ sin­(2Φ) was added in eq 4, and we have

5 $$V_{A1}=A_{\mathrm{FL},Y}\;\sin\left(2\Phi \right)\;\cos\left(\Phi \right)+A_{\mathrm{DL},Z}\;\sin\left(2\Phi \right)$$

*A*_DL,*Z*_ is the coefficient for the damping-like torque with spin polarizations along the *z* direction. The new formula (eq 5) shows a better fitting result (red curve in Figure 3d), which suggests the presence of an additional out-of-plane SOT component (see a more detailed analysis in Supplementary Note 1, Supplementary Figure S2, and Table S1). The in-plane (ξ_DL,*j*_^*Y*^) and out-of-plane (ξ_DL,*j*_^*Z*^) damping-like torque efficiencies per unit current density could be extracted using

6 $$\xi_{\mathrm{DL},j}^{Y}=\frac{S_{\mathrm{DL},Y}}{A_{\mathrm{FL},Y}}\frac{e\mu_{0}M_{S}t_{\mathrm{Pt}}t_{\mathrm{FM}}}{\hbar }\sqrt{1+\frac{M_{\mathrm{eff}}}{H_{R}}}$$

7 $$\xi_{\mathrm{DL},j}^{Z}=\frac{A_{\mathrm{DL},Z}}{A_{\mathrm{FL},Y}}\frac{e\mu_{0}M_{S}t_{\mathrm{Pt}}t_{\mathrm{FM}}}{\hbar }$$

The in-plane and out-of-plane damping-like torque efficiencies of θ_SH,*Y*_ = 0.147 ± 0.015 and θ_SH,*Z*_ = −0.013 ± 0.005 are obtained for spin polarization *S*_*y*_ and *S*_*z*_, respectively. Correspondingly, the effective spin Hall conductivity components are evaluated to be σ_SH,*Y*_ = 3.68 × 10^5^ (ℏ/2e) (Ω m)^−1^ and σ_SH,*Z*_ = −0.33 × 10^5^ (ℏ/2e) (Ω m)^−1^, respectively, which are comparable with TaIrTe_4_ ^43,44^ and PtTe_2_/WTe_2_ ^45^ and larger than WTe_2_.^38^ Interestingly, such an unconventional out-of-plane damping-like torque component is observed in the CFGT/Pt heterostructure using ST-FMR measurements.

To further verify this unconventional torque, we performed second harmonic Hall measurements in microfabricated Hall bar devices (see Figure 4a and the Methods section). Specifically, the in-plane angle dependence harmonic Hall measurement is used to extract the effective spin–orbit field H_DL(FL)_ along *x*-, *y*-, and *z*-axis directions and thermal contributions *V*_th_ with the 2^nd^ harmonic Hall voltage

8 $$V_{\mathrm{xy}}^{2\omega }=D_{\mathrm{DL},Y}\;\cos\left(\Phi \right)+D_{\mathrm{DL},Z}\;\cos\left(2\Phi \right)+F_{\mathrm{FL},Y}\;\cos\left(2\Phi \right)\;\cos\left(\Phi \right)+F_{\mathrm{FL},Z}$$

where

9 $$D_{\mathrm{DL},Y}=-\frac{H_{\mathrm{DL},Y}V_{\mathrm{AHE}}}{2\left(H+Hk\right)}+V_{\mathrm{th}}$$

10 $$D_{\mathrm{DL},Z}=-\frac{H_{\mathrm{DL},Z}V_{\mathrm{PHE}}}{H}$$

11 $$F_{\mathrm{FL},Y}=-\frac{H_{\mathrm{FL},Y}V_{\mathrm{PHE}}}{H}$$

**Figure 4:**
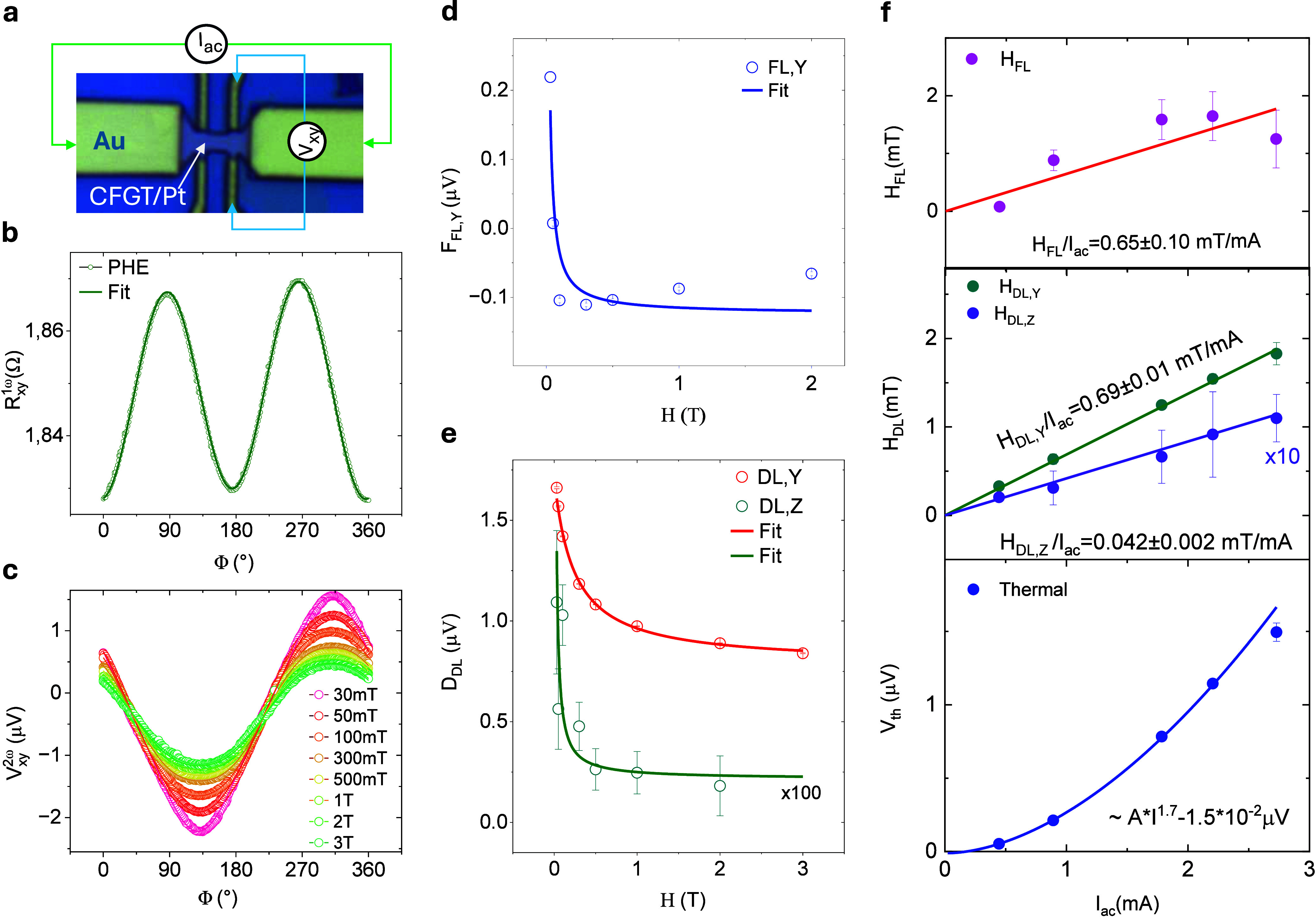
Harmonic measurements of the CFGT/Pt heterostructure at room temperature. (a) Schematics of the harmonic measurement setup with CFGT­(15 nm)/Pt­(10 nm) heterostructure Hall bar device. (b) First harmonic signal as a function of the in-plane angle with *H*_ip_ = 1 T and fitting result (solid curve). (c) In-plane angle dependence of the 2^nd^ harmonic signals with different fields and corresponding fitting (solid) curves at *I*_ac_ = 1.78 mA. (d and e) Extracted effective *H*_FL_ and *D*_DL_ components as a function of the external field with fitting results. (f) Extracted effective field *H*_FL_, *H*_DL_, and thermal contribution as a function of the bias currents. The solid curves are the linear and nonlinear fitting results.

Φ is the angle between the applied current *I*_ac_ and the magnetic field *H*. *V*_AHE_ and *V*_PHE_ are the AHE voltage and planar Hall (PHE) voltage, respectively. *V*_th_ is the thermal-related contribution, like anomalous Nernst and spin Seebeck effects, and *H*_k_ is the magnetic anisotropy field. By measuring the in-plane angle dependence of the first harmonic signal *V*_*xy*_^1ω^ at a fixed large in-plane field, we can extract the *V*_PHE_ with *R*_*xy*_^1ω^ = *R*_PHE_ sin­(2Φ) + *R*_AHE_ cos­(Φ) + *c* (as shown in Figure 4b). The 2^nd^ harmonic signal *V*_*xy*_^2ω^ in Figure 4c can be fitted with eq 8. The extracted components *F*_FL,*Y*_ and *D*_DL,*Y*(*Z*)_ as a function of the external field are plotted in Figure 4d,e. To further evaluate the effective fields induced by the SOT, eqs 9–11 are adopted, respectively. The bias dependence of the effective fields and thermal contribution are presented in (Figure 4f), which exhibit strong linear and parabolic relations with the bias current, respectively.

Because the damping-like torque is attributed to the longitudinal spin–orbit effective field *H*_DL_, the spin–orbit efficiency can be calculated.^46,47^

12 $$\xi_{\mathrm{DL},Y\left(Z\right)}=T_{\mathrm{ini}}\theta_{\mathrm{SH}}=\frac{2e}{\hbar }\mu_{0}M_{S}t_{\mathrm{CFGT}}^{\mathrm{eff}}H_{\mathrm{DL},Y\left(Z\right)}/J_{\mathrm{ac}}$$

where *J*_ac_ is the current density of Pt, e is the electron charge, ℏ is the reduced Planck constant, and μ_0_ *M*_S_ and *t*_CFGT_^eff^ are the saturation magnetization and the effective thickness of CFGT, respectively. By assuming the fully transparent interface, i.e., *T*_ini_ = 1, and effective saturation magnetization μ_0_ *M*_S_ = μ_0_ *M*_eff_, we obtain spin Hall angle θ_SH,*Y*_ = 0.157 ± 0.002 and σ_SH,*Y*_ = (3.93 ± 0.05) × 10^5^ (ℏ/2e) (Ω m)^−1^ and θ_SH,*Z*_ = 0.010 ± 0.001 and σ_SH,*Z*_=(0.23 ± 0.03) × 10^5^ (ℏ/2e) (Ω m)^−1^, which are comparable to the results obtained from ST-FMR experiments.

Our study of mechanisms governing magnetization dynamics and SOT in vdW magnet CFGT with in-plane magnetic anisotropy at room temperature uncovers their unique and unconventional spin–orbit torque behavior. Both ST-FMR and second harmonic Hall measurements show that the SOT efficiency of CFGT/Pt along S_*y*_ polarization is comparable to that of the other vdW magnets in a heterostructure with Pt.^25,30–33,48^ However, in addition, a large unconventional SOT component *S*_*z*_ is also observed. Our control experiments of a single CFGT nanolayer with second harmonic Hall measurements prove that the self-torque from the single CFGT nanolayers can be ruled out (see detailed results in Supplementary Note 2 and Supplementary Figure S3). Furthermore, Pt is a well-studied SOT material with a dominant *S*_*y*_ component, so any bulk material-related unconventional SOT effects can be ruled out. Furthermore, our previous study on CFGT/graphene^22^ suggests that the in-plane magnetic anisotropy of CFGT is independent of thickness variation. Therefore, the unconventional spin component should not originate from bulk CFGT, but rather from an interface-driven phenomenon. To confirm this, our DFT calculations combined with Monte Carlo simulation show that proximity with Pt introduces complex noncolinear spin textures in the CFGT (Figure 5a,b), which can be attributed to the strongly enhanced Dzyaloshinsky–Moriya interaction (Supplementary Figures S5 and S6), and weakened magnetic anisotropy energy with tilted magnetic easy axis (∼16°; Figure 5c and Supplementary Figure S4). This is in contrast to the pristine CFGT, which exhibits in-plane magnetic anisotropy, consistent with our previous experimental results (see a more detailed discussion in Supplementary Note 3). These observations suggest that the reported unconventional torque is not specific to a particular thickness or a single surface but is instead a robust feature of the CFGT/Pt interface.

**Figure 5:**
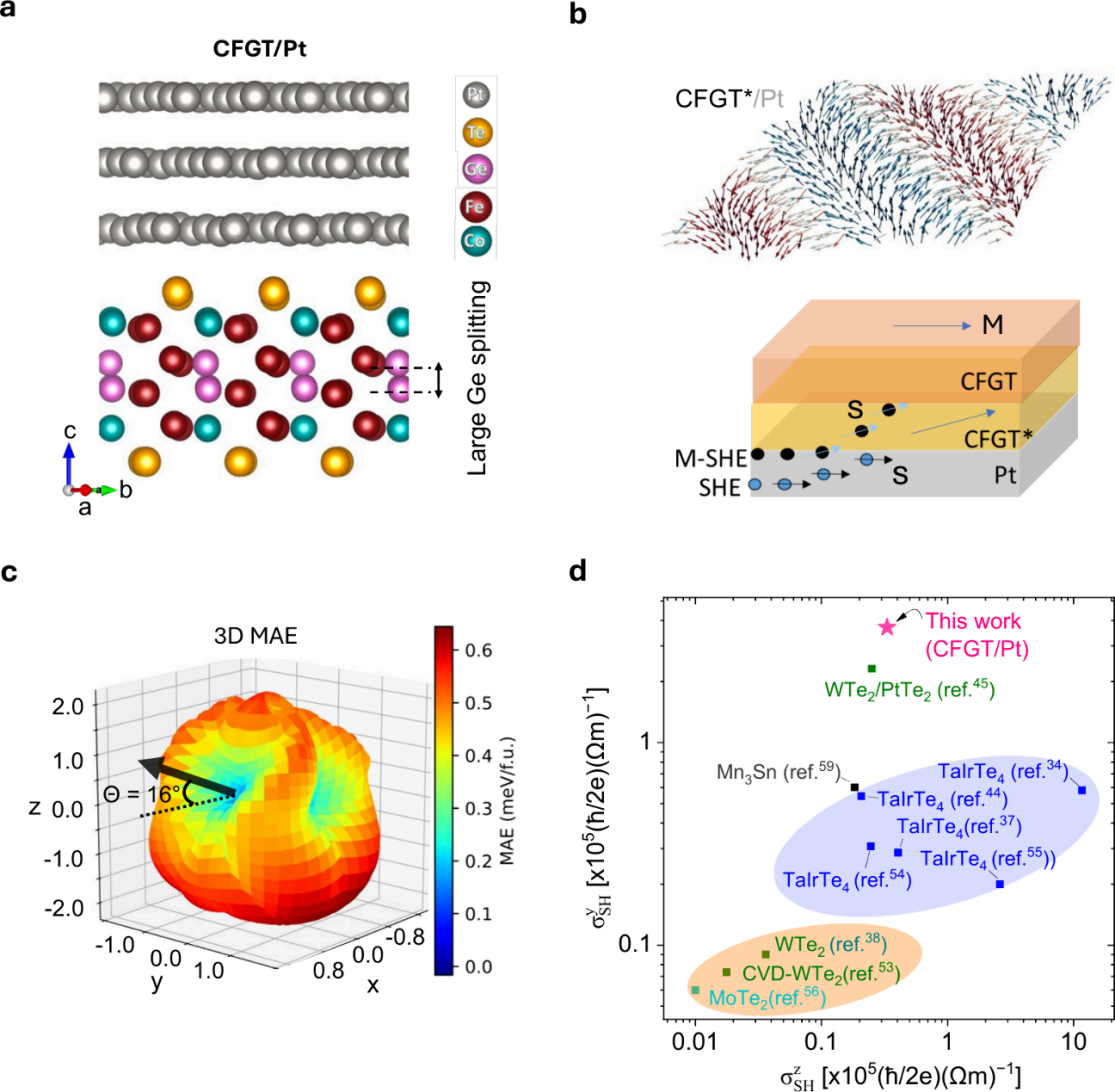
Theoretical calculations: DFT and Monte Carlo simulation results for CFGT/Pt heterostructure. (a) Schematic illustration of the relaxed geometry of CFGT/Pt atomic crystal structures. (b) Top panel: Monte Carlo simulation of the magnetic spin textures of CFGT/Pt. Bottom panel: Schematic illustration of the spin Hall effect (SHE) induced in-plane spin polarization and titled magnetic dependent spin Hall effect induced the unconventional spin component in modified CFGT interfaced with Pt. (c) 3D magnetic anisotropy energy (MAE) of CFGT/Pt. (d) State-of-the-art spin Hall conductivity in representative SOT devices with multiple spin polarizations.

Although such interface-related spin reorientation phenomena in conventional ferromagnetic materials heterostructures have been reported before, the origin remains elusive.^49–51^ Here, we show that the modified magnetic anisotropy and interfacial DMI at the CFGT/Pt interface can induce the spin reorientation toward an unconventional spin polarization, in addition to the traditional spin Hall effect-induced counterpart (Figure 5c, bottom panel). This largely expands the library for multiple spin polarization mechanisms (Figure 5d), including (1) lower crystal symmetry related SHE (such as WTe_2_,^38,52,53^ TaIrTe_4_,^34,37,38,44,54,55^ and MoTe_2_ ^56^), (2) spin swapping effect,^45^ which converts the in-plane spin polarization to the out-of-plane one due to crystal asymmetry, (3) broken time-reversal symmetry (ferromagnetic moments) related effects, such as the anomalous spin Hall effect.^57–59^

We further compare the magnetization dynamics parameters of CFGT with other vdW magnets (like Fe_*x*_GeTe_2_)^60,61^ and conventional magnetic thin films (Ni_80_Fe_20_, CoFeB).^62–65^ The CFGT damping constant is lower than that of other vdW magnets, while relatively higher than that of conventional magnetic thin films. However, vdW magnets, due to their layered nature, could host a larger magnetic anisotropy *H*_k_ without dealing with the traditional thin films’ complicated interface engineering.^64,65^ In particular, CFGT exhibits strong in-plane anisotropy and a relatively lower damping constant α at room temperature, which has high potential for magnetization dynamic applications. It reveals a previously unexplored symmetry-breaking mechanism at the CFGT/Pt interface and expands the understanding of how different torque components can emerge in vdW heterostructures. These findings are expected to be highly relevant for future studies aiming to engineer magnetic anisotropy, enable deterministic switching in related systems, or utilize such unconventional torques in other magnetic configurations. Moreover, although out-of-plane magnets are preferred for the high-density SOT magnetic random-access memory (MRAM) technology to replace the high capacity of dynamic random-access memory (RAM) cells, however, in-plane magnets with larger endurance and faster magnetization dynamics should be sufficient to replace static RAM and cache memory applications, where speed is more important than density.

In conclusion, we observed multidirectional magnetization dynamics with a significant SOT efficiency using vdW magnet CFGT in a heterostructure with Pt at room temperature. Both ST-FMR and second harmonic Hall experiments reveal large charge-to-spin conversion and damping-like SOT efficiencies, with observation of an unconventional out-of-plane spin Hall conductivity, an effect distinct from conventional ferromagnet/Pt heterostructures. This unconventional torque component, which manifests as an additional damping-like torque with out-of-plane spin polarization, is expected to originate from interface-driven spin reorientation effects, due to strong interfacial DMI and modified MAE landscape. The extracted magnetization dynamics parameters, effective magnetization of 0.321 T and Gilbert damping constant of 0.027, position CFGT as a competitive platform for spintronic devices. These findings on spin dynamics in vdW magnetic heterostructures indicate that interface engineering may enable pathways to nonvolatile spintronic devices with enhanced functionality.^66^

## Methods

### Device Fabrication

The van der Waals crystals of CFGT were grown by HqGraphene. The CFGT samples were prepared by mechanically exfoliating onto a SiO_2_/Si wafer using the Scotch tape method inside a glovebox. Pt was deposited by electron beam evaporation after 15 s of Ar plasma cleaning to fabricate CFGT/Pt heterostructures. The CFGT/Pt heterostructures were then patterned into the rectangular microstrips for ST-FMR measurements and Hall bars for harmonic measurements using the Ar ion milling with e-beam resist as the etching mask.

### Anisotropic Magnetoresistance Measurements

In-plane angular dependence, anisotropic magnetoresistance (AMR) measurements are performed on microbars using a rotatable projected vector field magnet with a fixed applied magnetic field of 1 T and applied DC current of 0.5 mA. The resistance of the devices was measured while rotating the magnetic field in the plane.

### Spin Torque Ferromagnetic Resonance (ST-FMR) Measurements

ST-FMR measurements are performed at room temperature on the microbar devices to estimate the spin–orbit torque (SOT) efficiency and other magnetodynamical parameters. The measurements for effective SOT analysis are performed with a fixed in-plane angle ϕ = 60°, the radio-frequency (RF) current modulated at 98.76 Hz is applied to the device through a high-frequency bias-T at a fixed frequency (ranging from 3 to 14 GHz) with an input RF power *P* = 4 dBm. The in-plane angle ϕ dependence ST-FMR measurements are performed using a rotatable projected field magnet with ϕ = 0–360° (step of 15°) at a fixed frequency.

### 2nd Harmonic Hall measurements

The 2^nd^ harmonic measurements in the high magnetic field range were carried out in the Quantum Design DynaCool PPMS system with an external electronic connection to Lockin SR830 to measure the first and second harmonic voltages with fixed frequency ω = 213.34 Hz.

## Supplementary Material



## Data Availability

The data supporting the findings of this study are available from the corresponding authors upon reasonable request.

## Notes

Analysis of ST-FMR results (Supplementary Note 1), control experiments for self-torque in CFGT (Supplementary Note 2), magnetic dynamic parameters of this work at room temperature (Supplementary Table S1), and DFT supplemental results (Supplementary Note 3) (PDF)
