# Supplementary material for: Multidirectional Spin–Orbit Torque Magnetization Dynamics in beyond Room Temperature Van der Waals Magnet Devices
Source: Nano Lett. 2026 Jul 14;26(29):9631–40. doi: 10.1021/acs.nanolett.6c02494 (PMC13430683; doi:10.1021/acs.nanolett.6c02494)
Supplement: Supplementary file 1 [file nl6c02494_si_001.pdf]

Supplementary information

Multi-directional spin-orbit torque magnetization dynamics in beyond room temperature van der Waals magnet devices

Bing Zhao[1†*], Lakhan Bainsla[1,2†], Soheil Ershadrad[3,4], Prabhav N. Sumant[1], Roselle Ngaloy[1], Johanna Rosen[3,4], Biplab Sanyal[5], Johan Åkerman[6,7,8], Saroj P. Dash[1,9,10*]

[1]Department of Microtechnology and Nanoscience, Chalmers University of Technology, SE-41296, Göteborg, Sweden.
[2]Department of Physics, Indian Institute of Technology Ropar, Roopnagar 140001, India.
[3]Department of Physics, Thin Film Physics, Chemistry and Biology (IFM), Linköping University, Linköping, SE-581 83, Sweden.
[4]Wallenberg Initiative Materials Science for Sustainability (WISE), Department of Physics, Chemistry and Biology (IFM), Linköping University, Linköping 58183, Sweden.
[5]Department of Physics and Astronomy, Uppsala University, Box 516, Uppsala SE-751 20, Sweden.
[6]Department of Physics, University of Gothenburg, SE-41296, Göteborg, Sweden.
[7]Center for Science and Innovation in Spintronics, Tohoku University, 2-1-1 Katahira, Aoba-ku, Sendai 980-8577 Japan.
[8]Research Institute of Electrical Communication, Tohoku University, 2-1-1 Katahira, Aoba-ku, Sendai 980-8577 Japan.
[9]Wallenberg Initiative Materials Science for Sustainability, Chalmers University of Technology, SE-41296, Göteborg, Sweden.
[10]Graphene Center, Chalmers University of Technology, SE-41296, Göteborg, Sweden.

Content:

1. *Supplementary Note 1. Analysis of ST-FMR results*
2. *Supplementary Note 2. Control experiments for self-torque in CFGT*
3. *Supplementary Table S1. Magnetic dynamic parameters of this work at room temperature*
4. *Supplementary Note 3. Density functional theory (DFT) supplemental results.*

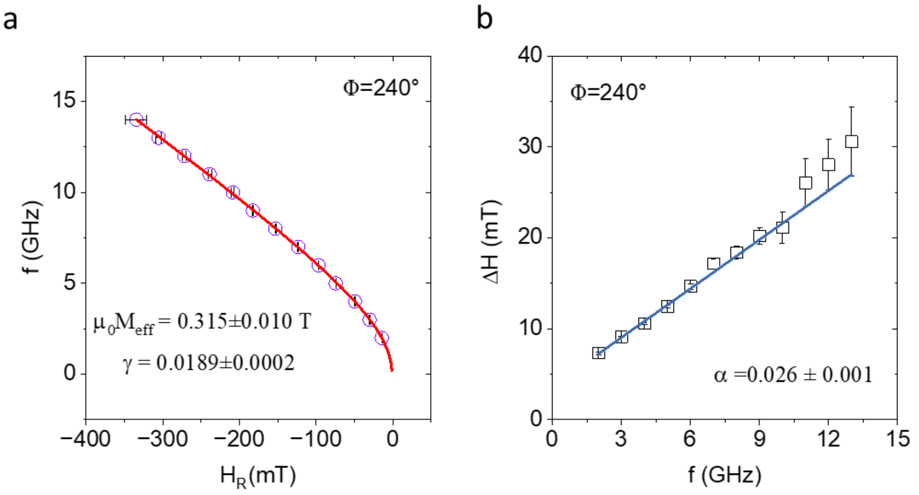

Supplementary Figure S1. *a.* *Fitting of resonant frequency f v.s. the extracted resonant field H_R at Φ=240°.* ***b.*** *Fitting of resonant extracted linewidth ΔH v.s. resonant frequency f. All the solid curves are the fitting results. The error bars are calculated from the standard deviation of the signal background. All the measurements are from the ST-FMR device.*

Supplementary Note 1. Analysis of ST-FMR results

To analyze this phenomenon in more detail, we performed the angular dependent STFMR measurements, and representative curves for sample CFGT/Pt are shown in the main text Fig. **3**. The S and A values are extracted with different ϕ values and their angular dependence are plotted as shown in Fig. **3c** and **3d**, respectively. In general, the spin current that generates torque has components along all of the x, y, and z axes, thus the allowed angular dependencies for the coefficients V_S and V_A[1–4],

$$V_S = S_{DL,Y}\cos\phi\sin 2\phi + S_{DL,X}\sin\phi\sin 2\phi + S_{FL,Z}\sin 2\phi; \quad \text{(Eq. S1)}$$

$$V_A = A_{FL,Y}\cos\phi\sin 2\phi + A_{FL,X}\sin\phi\sin 2\phi + A_{DL,Z}\sin 2\phi; \quad \text{(Eq. S2)}$$

where $S_{DL,Y}$, $S_{DL,X}$, and $A_{DL,Z}$ are the coefficients for the damping-like torque with spin polarizations along the x, y, and z axes, respectively. $A_{FL,Y}$, $A_{FL,X}$, and $S_{FL,Z}$ are field-like torques with polarization along different axes. The angular dependence V_S and V_A is fitted using Eq. S1 and Eq. S2 and the obtained parameters from the fitting are given in Supplementary Table 1. Note that the V_A component is fitted with Eq. S2 with different spin polarizations as a comparison (Fig. S2).

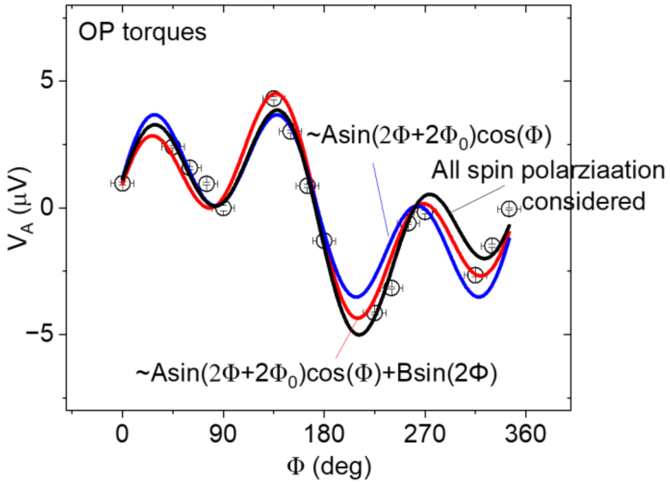

Supplementary Figure S2. *Angle dependence of the extracted anti-symmetric component* V_A. *Solid curves denote the fitting to the data using equations indicated in the figure.*

Supplementary Note 2. Control experiments for self-torque in CFGT

To further evaluate the CFGT self-torque without a Pt layer, we also fabricate a CFGT Hall bar device with an AlO_x capping layer. Firstly, the CFGT shows the in-plane magnetism with both IP and OP AHE signals (Fig. S6). Then, the 2nd harmonic Hall measurements were performed to extract the effective SOT components. Different methods were adopted as below, where S_y, S_x, and S_z are considered with different combinations (also see Eq. 8 in the main text)[5,6]

$$V^{2\omega}_{xy}=D_{DL,Y}\cos(\Phi)+F_{FL,Y}\cos(2\Phi)\cos(\Phi); \quad \text{(Eq. S3)}$$

$$V^{2\omega}_{xy}=D_{DL,Y}\cos(\Phi)+D_{DL,X}\sin(\Phi)+D_{DL,Z}\cos(2\Phi)+F_{FL,Y}\cos(2\Phi)\cos(\Phi)+F_{FL,X}\cos(2\Phi)\sin(\Phi)+F_{FL,Z}; \quad \text{(Eq. S4)}$$

The extracted effective components $D_{DL,Y(Z)}$, and F_{FL} as functions of the field are presented in Fig. S3c-Fig. S3e. However, it is not possible to fit and extract the effective fields with Eq. 9-Eq. 11 from the main text. These suggest that the CFGT self-torques are very small to extract any reasonable parameters and are ignorable compared to those of CFGT/Pt devices.

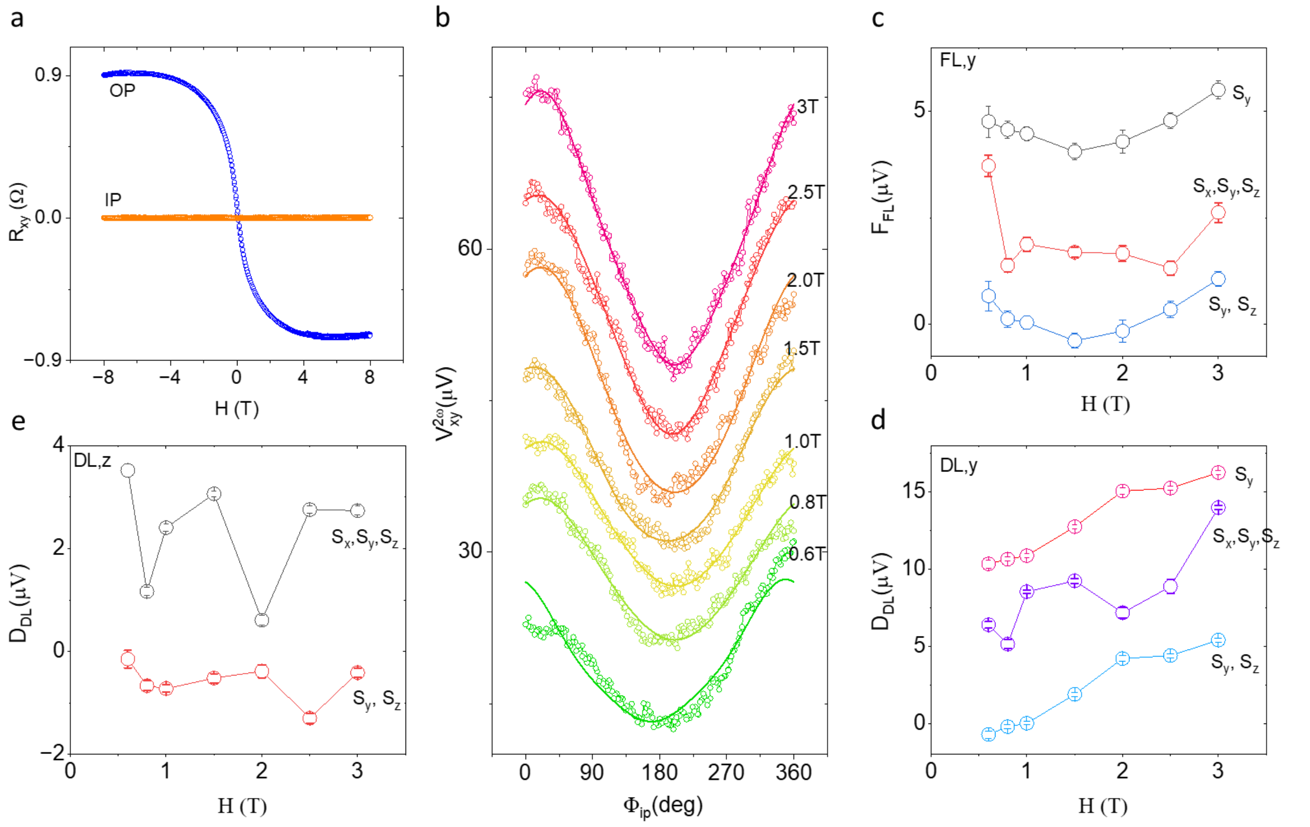

Supplementary Figure S3. Self-torque evaluation in only CFGT using 2nd harmonic measurements. a. AHE *signal of CFGT with out-of-plane* (OP) *and in-plane* (IP) *magnetic field.* ***b.*** *In-plane angle dependence of the 2nd harmonic signals with different fields and corresponding fitting (solid) curves.* ***c-e.*** *Extracted effective* F_{FL} *and* $D_{DL,Y(Z)}$ *components as a function of the external field at* I_{ac}=1 *mA.*

Supplementary Table S1. Magnetic dynamic parameters of this work at room temperature.

The CFGT(t_{CFGT})/Pt (10 nm) devices for ST-FMR and 2nd harmonic Hall (SHH) measurements have the geometry parameters as below, aspect ratio ξ=L/W, RF current and magnetic field angle Φ, damping constant α, effective magnetization $\mu_0 M_{eff}$, resonance field H_R, and spin Hall conductivity $\sigma_{SH}=\theta_{SH}/\rho_{Pt}$, ($\rho_{Pt}=4\times10^{-7}$ Ωm, $\rho_{CFGT}=1.05\times10^{-6}$ Ωm).

Methods	t_{CFGT} (nm)	W (µm)	ξ= L/W	Φ (deg)	α	γ	$\mu_0 M_{eff}$ (T)	H_R (T) (f=5 GHz)	$\delta\Delta H/\delta I_{dc, Pt}$ (mT/mA)	$S_{DL,Y}$ (µV)	$A_{FL,Y}$ (µV)	$A_{DL,Z}$ (µV)
ST-FMR	25	1.8	3:1	60	0.027(1)	0.0187(2)	0.321±0.014	0.086	1.568±0.141	8.439±0.402	4.668±0.487	-0.889±0.310
				240	0.026(1)	0.0189(2)	0.315±0.010	0.073	-0.967±0.107			
Methods	t_{CFGT} (nm)	W (µm)	H_k (T)			$\Delta H_{DL,Y}/J_{ac}$ (mT/MA/cm²)	$\theta_{SH,Y}$	$\sigma_{SH,Y}$ (ħ/2e) (Ωm)$^{-1}$		$\Delta H_{DL,Z}/J_{ac}$ (mT/MA/cm²)	$\theta_{SH,Z}$	$\sigma_{SH,Z}$ (ħ/2e) (Ωm)$^{-1}$
SHH	15	1.4	0.24			0.135±0.002	0.157±0.002	(3.93±0. 05) x10^5		0.009±0.002	0.010±0.001	(0.23±0.02) x10^5

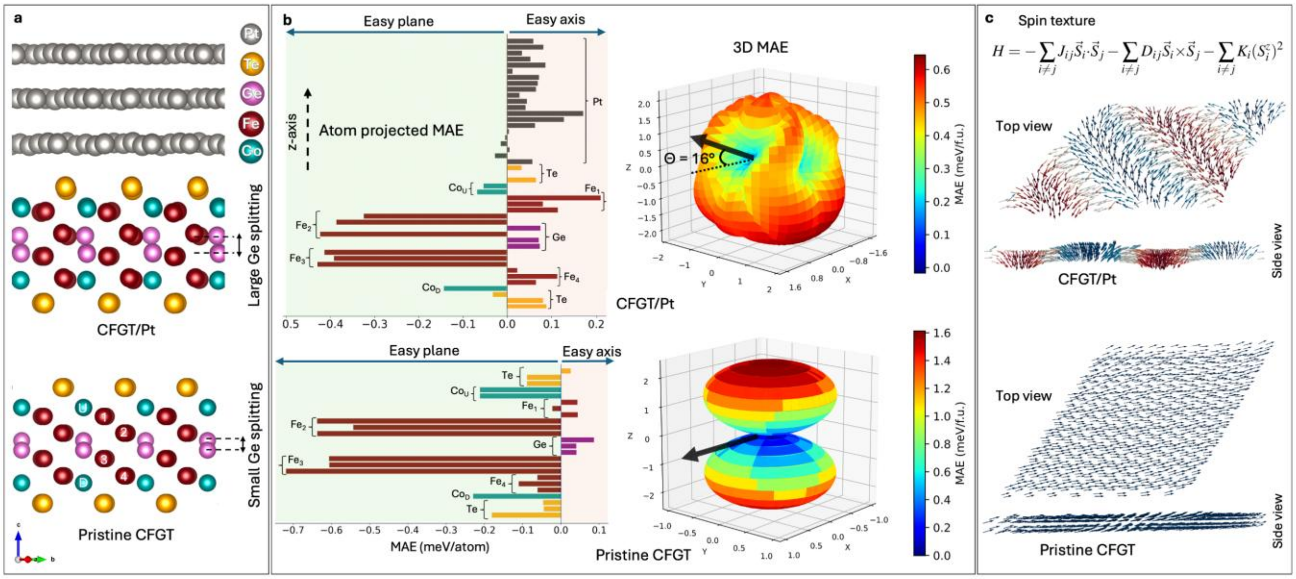

Supplementary Figure S4. *DFT and Monte Carlo Simulation Results: A Comparison between CFGT/Pt (upper panels) and Pristine CFGT (lower panels) (a) Schematic illustration of the relaxed atomic crystal structures of CFGT/Pt and pristine CFGT. (b) Three-dimensional magnetic anisotropy energy (MAE) and atom-resolved MAE for CFGT/Pt and pristine CFGT. (c) Spin textures obtained from Monte Carlo simulations for CFGT/Pt and pristine CFGT.*

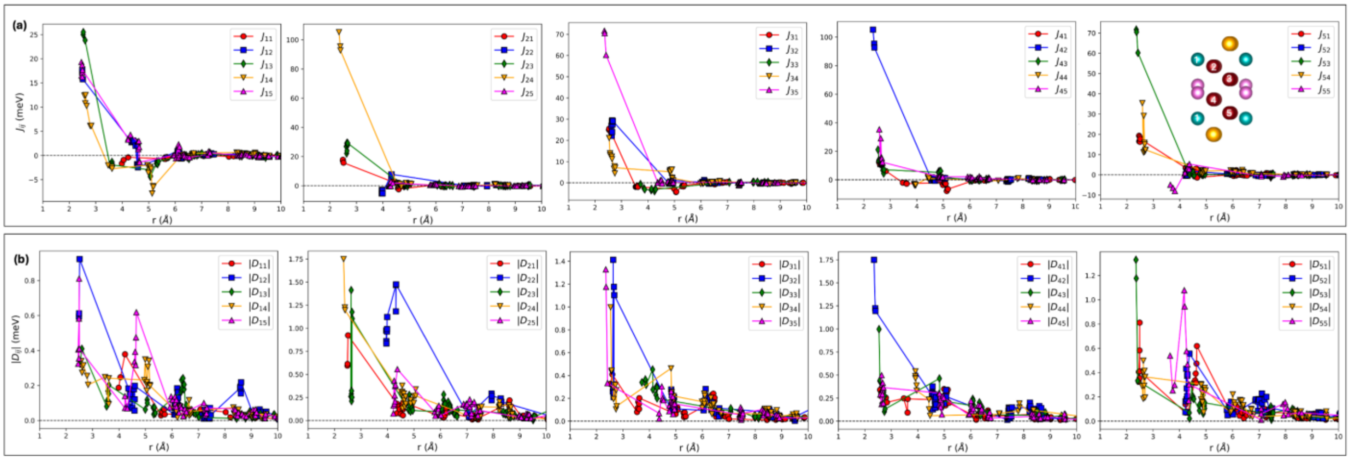

Supplementary Figure S5. *(a) Isotropic Heisenberg exchange interactions (J_{ij}) and (b) norm of the DMI vectors in CFGT/Pt, where the indices i and j iterate over the sublattices labeled in the schematic crystal structure.*

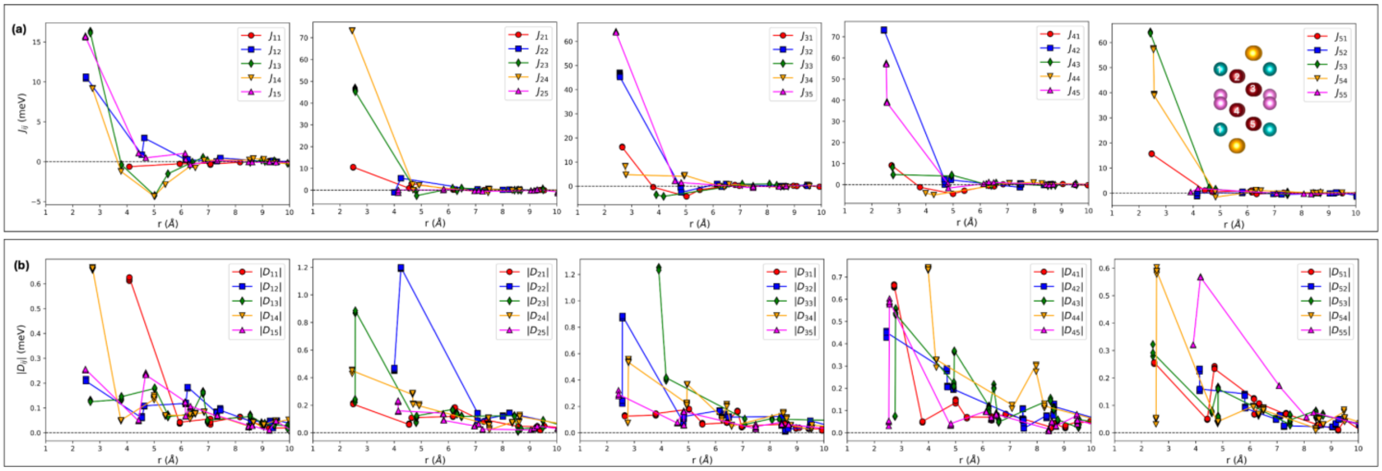

Supplementary Figure S6. *(a) Isotropic Heisenberg exchange interactions (J_{ij}) and (b) norm of the DMI vectors in pristine CFGT, where the indices i and j iterate over the sublattices labeled in the schematic crystal structure.*

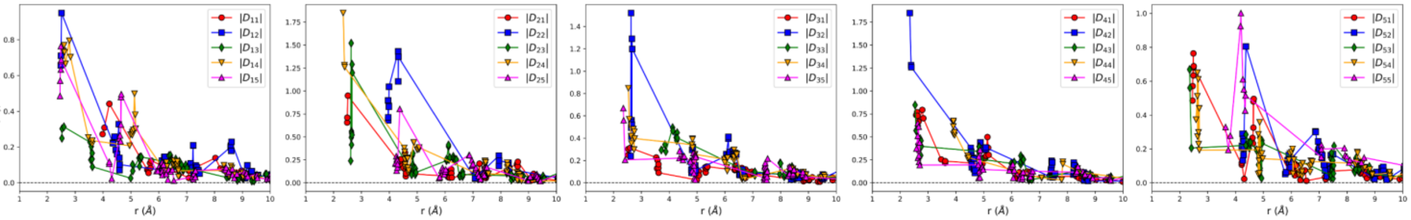

Supplementary Figure S7. *Norm of DMI in deformed CFGT in the absence of Pt layer.*

Supplementary Note 3. Density functional theory (DFT) supplemental results

1. Heterostructure and Structural Relaxation - Structural relaxation was performed on a √3×√3 R30° supercell of FGT doped with 20% Co (owing to supercell size constraints) at the outermost Fe sublayer, substituting Fe atoms with Co. The outermost sublayer was selected based on our earlier studies demonstrating that it is the most energetically favorable site for Co doping. The energetically favorable up-down-up (UDU) configuration was adopted for the distribution of Co atoms[7–9]. The optimized CFGT structure was subsequently interfaced with three sublayers of Pt along the (111) surface (√7×√7 R19.1°), resulting in ~3% lattice strain in the heterostructure. To identify the most energetically favorable interface configuration, the Pt film was systematically shifted laterally along the x- and y-axes. The optimized structure in POSCAR format is provided at the end of Supplementary Note 3.

2. Origin of interfacial effects - A comparison of the schematic crystal structures of CFGT/Pt and pristine CFGT is presented in Supplementary Figure S4(a). It is evident that proximity to Pt introduces structural deformation into the adjacent CFGT layer. This deformation is well reflected in the Ge splitting intrinsic to FGT and its doped variants[7], which is found to be larger on average in CFGT interfaced with Pt. These observations suggest that the interfacial effects between CFGT and Pt arise from two primary origins: charge transfer between Pt and CFGT, and structural deformation induced by Pt proximity.

3. Physical Properties Modified by Interfacial Effects - To understand how proximity-induced effects alter the magnetic properties of CFGT, three key magnetic features governing spin texture formation were systematically studied and compared: magnetic anisotropy energy (MAE), isotropic Heisenberg exchange interactions (J_{ij}), and the Dzyaloshinskii–Moriya interaction (DMI). Although other factors, such as higher-order exchange interactions and dipole–dipole interactions, may also be modified in heterostructures, their contributions are generally negligible compared to those of the aforementioned features[10].

It was found that the MAE landscape of CFGT is significantly affected by Pt proximity. Supplementary Figure S4(b) compares the 3D MAE landscape and atom-resolved MAE in CFGT/Pt and pristine CFGT. It is evident that the 3D MAE landscape of pristine CFGT exhibits a toroidal shape, with an easy plane lying in the xy-plane (i.e., the easy axis can adopt any orientation within the xy-plane), and a MAE of 1.61 meV/f.u. (i.e., the energy difference between the easy and hard axes). In CFGT/Pt, however, the 3D MAE landscape becomes asymmetric, where the easy axis is no longer confined to the xy-plane but instead exhibits a tilting of approximately 16° toward the z-axis. Furthermore, the MAE is significantly reduced to 0.64 meV/f.u. Although strong spin-orbit coupling (SOC) in Pt is generally expected to enhance the MAE of the heterostructure, it is known that orbital hybridization between different states (in this case, Pt and those of the adjacent layer) can act to partially cancel the MAE contribution[11], as observed here in CFGT/Pt.

The atom-resolved MAE more clearly reveals the origin of the MAE reduction, where negative values indicate an easy-plane tendency and positive values indicate an easy-axis tendency perpendicular to the plane. It is evident that in pristine CFGT, the dominant easy-plane tendency originates from the central Fe2 and Fe3 sublattices. In the presence of Pt, however, not only do the Pt atoms favor a perpendicular easy axis, but the easy-plane tendency (negative values) of all Fe and Co sublattices is also substantially diminished, with some of the sublayers closest to Pt even reversing their sign.

Supplementary Figures S5 and S6 compare the isotropic Heisenberg exchange interactions (J_{ij}) (upper panels) and DMI (lower panels) for CFGT/Pt and pristine CFGT, respectively, where the

indices i and j are iterated over sublayers rather than individual atoms for clarity of visualization. It is evident that in both CFGT/Pt (strongest J_{24} = 105 meV) and pristine CFGT (strongest J_{24} = 73 meV), the nearest-neighbor exchange interactions are considerably strong ferromagnetic. As established in earlier studies, this is the driving force behind the above-room-temperature magnetic transition temperature in these systems[7]. On average, however, a systematic enhancement of the nearest-neighbor exchange interaction strength is observed in CFGT/Pt. This indicates, first, that CFGT/Pt should exhibit a higher Curie temperature (T_C) than pristine CFGT and, second, given that exchange interaction strength is among the key factors governing spin texture formation, the enhanced exchange interactions in the presence of Pt are expected to further modify the spin texture of CFGT.

Comparison of the DMI plots reveals two notable features. First, the structural deformation induced by proximity to Pt further breaks the crystalline symmetry and thus lifts the degeneracy of the DMI vectors (i.e., a greater number of non-overlapping DMI points are visible in the CFGT/Pt plots). This indicates that the DMI vectors do not cancel each other effectively in CFGT/Pt, giving rise to a finite net spiralization tendency, whereas in pristine CFGT, this tendency is significantly suppressed. Second, the DMI strength is also enhanced on average in CFGT/Pt relative to pristine CFGT; for example, the norm of the nearest-neighbor D_{42} increases from approximately 0.4 meV to approximately 1.75 meV in the presence of Pt. To quantify these differences, the micromagnetic spiralization tensor was calculated for both CFGT/Pt and pristine CFGT using the following expression,

$$D_{\alpha\beta} = \Sigma_{(j\neq i)} D_i^{\alpha} j R_i^{\beta} j e^{(-\mu R_i j)}; \qquad \text{(Eq. S5)}$$

where $D_{ij}^{\alpha}$is the DMI vector component between sites $i$and j, and $R_{ij}$is the intersite distance. The parameter $\mu$was varied in the range of 2–4 and subsequently extrapolated to μ= 0 by fitting a third-order polynomial, thereby improving numerical convergence with respect to the real-space cutoff. For a heterostructure of the form of CFGT/Pt, the spiralization tensor takes the following form,

$$\begin{bmatrix} 0 & D & 0 \\ -D & 0 & 0 \\ 0 & 0 & 0 \end{bmatrix}$$

where the scalar Ddenotes the spiralization constant (see Ref. [12] for details). It was found that CFGT/Pt exhibits a spiralization constant of D= 13.02 meV·Å, compared to D= 3.84 meV·Å for pristine CFGT, indicating a higher possibility of chiral spin texture in CFGT/Pt.

4. Monte Carlo Simulations - The spin texture in CFGT/Pt and pristine CFGT was further simulated based on the magnetic features discussed above (see Supplementary Note 4 for methodological details). The resulting spin textures are compared in Figure S4(c). It is evident that while pristine CFGT exhibits a nearly collinear spin texture, CFGT/Pt displays a highly non-collinear and complex spin texture. The collinear spin texture in pristine CFGT primarily stems

from the strong easy-plane MAE, which constrains the magnetic moments within the xy-plane, in conjunction with a weak spiralization tendency arising from weak DMI. In contrast, CFGT/Pt not only possesses a significantly reduced MAE with a tilted easy axis but also exhibits a strong spiralization tendency originating from enhanced DMI. The interplay of these competing effects gives rise to the complex spin texture observed in CFGT/Pt. We propose that this complex magnetic behavior constitutes the primary physical mechanism underlying the strong anomalous SHC observed in this system.

5. Decoupling Structural Deformation and Charge Transfer Effects - As a controlled test to decouple the effects of structural deformation from charge transfer between the layers, analogous calculations were performed for the deformed CFGT structure with the Pt atoms removed. It was found that both the reduced MAE magnitude and the tilted easy axis originate primarily from charge transfer between Pt and CFGT, as these effects are diminished upon removal of the Pt atoms (No tilting of the easy axis was observed in the absence of Pt). This can be attributed to the absence of orbital hybridization between Pt and CFGT states. As discussed earlier, such hybridization constitutes the driving force behind the reduced MAE; consequently, removal of the Pt layer is expected to eliminate the aforementioned hybridization effects.

However, the same reasoning does not apply to the strength of the DMI, where the symmetry breaking introduced by structural deformation plays the dominant role in enhancing DMI in the presence of Pt (DMI of comparable magnitude was observed in deformed CFGT even after removing the Pt thin film, see Supplementary Figure S7).

Density functional theory (DFT) methodology

First-principles structural optimization was performed within the framework of density functional theory (DFT)[13,14] using the VASP[15-17]. The exchange-correlation interaction was described using the generalized gradient approximation (GGA) with the Perdew-Burke-Ernzerhof (PBE) functional[18]. The projector augmented-wave (PAW) method[19] was employed to describe the electron-ion interaction.

The electronic wave functions were expanded on a plane-wave basis set with a kinetic energy cutoff of 500 eV. To eliminate spurious interactions between periodic images, a vacuum layer of at least 20 $\AA$ was introduced along the c-axis. Structural optimization was considered converged when the residual force on each atom was below 1×10^{-2}eV/$\AA$. A Gaussian smearing width of 0.05 eV was applied throughout the calculations. The CFGT structure was constructed by selectively doping the outermost Fe sublattices in a $\sqrt{3} \times \sqrt{3}$ supercell of FGT, corresponding to the energetically most favorable configuration, as discussed in detail in Refs.[7-9]. Brillouin-

zone integrations were carried out using a Γ-centered $7 \times 7 \times 1$Monkhorst-Pack k-point mesh for the monolayer systems.

For the magnetic calculations, spin-orbit coupling (SOC) was explicitly included. The Heisenberg exchange interactions, Dzyaloshinskii-Moriya interaction (DMI), magnetic anisotropy energy (MAE), and atom-resolved MAE were calculated using the QuantumATK package (Synopsys, version U-2022). The calculations employed a linear combination of atomic orbitals (LCAO) basis set together with the PseudoDojo pseudopotentials[20, 21], a density mesh cutoff energy of 120 Hartree, and a $15 \times 15 \times 1$ k-point mesh. The exchange interactions were evaluated using the Liechtenstein-Katsnelson-Antropov-Gubanov (LKAG) formalism[22].

The extracted exchange interactions were incorporated into the following Heisenberg Hamiltonian:

$$H = -\sum_{i \neq j} J_{ij} \vec{S}_i \cdot \vec{S}_j - \sum_{i \neq j} D_{ij} \vec{S}_i \times \vec{S}_j - \sum_{i \neq j} K_i (S_i^z)^2; \qquad \text{(Eq. S6)}$$

where J_{ij} represents the isotropic exchange interaction, D_{ij} denotes the Dzyaloshinskii-Moriya interaction (DMI), and K_i corresponds to the single-ion magnetic anisotropy constant. The vectors S_i and S_j denote the spin moments at lattice sites i and j, respectively. Classical Monte Carlo (MC) simulations were subsequently performed using the UppASD package[23]. To ensure statistically reliable averages, five independent ensembles were simulated within a $32 \times 32 \times 1$ supercell under periodic boundary conditions.

Supplementary Table 2. The optimized structure in POSCAR format.

	CFGT-Pt Structure	
	1.0000000000000000	
7.0763042140449501	0.0000000000000000	0.0000000000000000
3.5381521070224751	6.1282592142698027	0.0000000000000000
0.0000000000000000	0.0000000000000000	35.0000000000000000
	Co Fe Ge Te Pt	
	3 12 3 6 21	
	Direct	
0.035673294401297	0.652315822390735	0.293851155386837
0.692319242764497	0.331527451179603	0.404493339978816
0.381830643344116	-0.018575012401703	0.406881189821616
0.034021914619054	0.008209802265708	0.294785078133662
0.684072467679155	0.653463971416313	0.298020610089299
0.389108625390699	0.302632110701620	0.297817807670253
0.039367225032324	0.332064640341580	0.330808272463248
0.732500937187697	0.964911454464702	0.330298475346062
0.339157911003934	0.660182811231451	0.330769077475948
0.036126060613688	0.009801007165277	0.361773784446103
0.672317598618268	0.652233381569737	0.366506119094465
0.400835528904739	0.288101970274378	0.366095048851837
0.040727338873826	0.339684865693003	0.399873138515879
0.735597870754020	0.961984785084938	0.397533514439091
0.330228942141749	0.660304899858295	0.398311927409799

0.034658147397473	0.643220462489029	0.362373549211869
0.697054827108039	0.325759238599385	0.335093187284287
0.377420591529259	-0.016155156811626	0.336662491604055
0.036516410034873	0.333200622609930	0.258778249141191
0.712478075028309	-0.021389019451745	0.258786696439453
0.358117060416664	0.657271100926348	0.259102533869980
0.031931887250997	0.003456348309945	0.432757188926716
0.717475164258351	0.654131461214893	0.437632476054401
0.365109304121891	0.329046196598154	0.437638215559430
0.714448695655022	0.451139729068125	0.503742381951359
0.571280645537260	0.166709333320216	0.503744520050935
0.427771304728916	0.881274247117474	0.500989185809189
0.285317724722909	0.596297515930646	0.500447588571405
0.142458956343769	0.308494981465492	0.504645203278646
0.000728201619231	0.022112862764426	0.505277898919477
0.856562384744894	0.738716803482703	0.503515247871975
0.697532983736248	0.655772656270081	0.573433366730800
0.555767540617503	0.370007609021739	0.572886513136158
0.412171077687888	0.082970716875326	0.572536708337423
0.271149496436227	0.796300264743783	0.570630320226155
0.127748924071441	0.512393524062598	0.573040517152817
-0.016035964016425	0.228765393676264	0.573410509775083
0.840036763772450	0.940591461054508	0.572271495880275
0.820369609841141	0.148386493715908	0.641420453284030
0.677033306841080	0.863386803413441	0.641290033286696
0.533294730117442	0.577603030319034	0.641373766526580
0.391280231083000	0.291506406769509	0.641352686485498
0.248446242643725	0.005400292982304	0.640520593058668
0.105561778888032	0.720562597844442	0.640341969805738
0.962427696453264	0.434221204382026	0.642619565655035

References

1. Liu, L., Moriyama, T., Ralph, D. C. & Buhrman, R. A. Spin-Torque Ferromagnetic Resonance Induced by the Spin Hall Effect. Phys. Rev. Lett. 106, 036601 (2011).

2. MacNeill, D. et al. Control of spin–orbit torques through crystal symmetry in WTe_2/ferromagnet bilayers. Nat. Phys. 13, 300–305 (2017).

3. Liu, Y. et al. Field-free switching of perpendicular magnetization at room temperature using out-of-plane spins from $TaIrTe_4$. Nat. Electron. 6, 732–738 (2023).

4. Bose, A. et al. Tilted spin current generated by the collinear antiferromagnet ruthenium dioxide. Nat. Electron. 5, 267–274 (2022).

5. Pandey, L. et al. Energy-efficient field-free unconventional spin-orbit torque magnetization switching dynamics in van der Waals heterostructures. arXiv 2408.13095 (2024).

6. Bainsla, L. et al. Large out-of-plane spin–orbit torque in topological Weyl semimetal $TaIrTe_4$. Nat. Commun. 15, 4649 (2024).

7. Ershadrad, Soheil, Sukanya Ghosh, Duo Wang, Yaroslav Kvashnin, and Biplab Sanyal. Unusual magnetic features in two-dimensional Fe_5GeTe_2 induced by structural reconstructions. The journal of physical chemistry letters 13, 4877-4883(2022).

8. Ghosh, Sukanya, Soheil Ershadrad, and Biplab Sanyal. Structural distortion and dynamical electron

correlation driven enhanced ferromagnetism in Ni-doped two-dimensional Fe_5GeTe_2 beyond room temperature. 2D Materials 11, 035002 (2024).

9. Ngaloy, Roselle, Bing Zhao, Soheil Ershadrad, Rahul Gupta, Masoumeh Davoudiniya, Lakhan Bainsla, Lars Sjostrom et al. Strong in-plane magnetization and spin polarization in $(Co_{0.15}Fe_{0.85})_5GeTe_2$/Graphene van der Waals Heterostructure Spin-Valve at Room Temperature. ACS nano 18, 5240-5248 (2024).

10. Szilva, Attila, Yaroslav Kvashnin, Evgeny A. Stepanov, Lars Nordström, Olle Eriksson, Alexander I. Lichtenstein, and Mikhail I. Katsnelson. Quantitative theory of magnetic interactions in solids. Reviews of Modern Physics 95, 035004 (2023).

11. Ershadrad, Soheil, Nikola Machacova, Arnob Mukherjee, Vladislav Borisov, Olle Eriksson, and Biplab Sanyal. Complex magnetic exchange, anisotropy and skyrmionic textures in 2D ferromagnets with transition metals and chalcogens. npj 2D Materials and Applications npj 2D Mater Appl 10, 46 (2026).

12. Borisov, Vladislav. From electronic structure to magnetism and skyrmions. Electronic Structure 6, 023002 (2024).

13. Kohn, Walter, and Lu Jeu Sham. Self-consistent equations including exchange and correlation effects. Physical Review 140, A1133 (1965).

14. Hohenberg, Pierre, and Walter Kohn. Inhomogeneous electron gas. Physical Review 136, B864 (1964).

15. Kresse, Georg, and Jürgen Furthmüller. Efficiency of ab-initio total energy calculations for metals and semiconductors using a plane-wave basis set. Computational materials science 6, 15-50 (1996).

16. Kresse, Georg, and Jürgen Hafner. Ab initio molecular-dynamics simulation of the liquid-metal–amorphous-semiconductor transition in germanium." Physical Review B 49, 14251 (1994).

17. Kresse, Georg, and Jürgen Hafner. Ab initio molecular dynamics for liquid metals. Physical Review B 47, 558 (1993).

18. Perdew, John P., Kieron Burke, and Matthias Ernzerhof. Generalized gradient approximation made simple. Physical Review Letters 77, 3865 (1996).

19. Blöchl, Peter E. Projector augmented-wave method. Physical Review B 50, 17953 (1994).

20. Smidstrup, Søren, Troels Markussen, Pieter Vancraeyveld, Jess Wellendorff, Julian Schneider, Tue Gunst, Brecht Verstichel et al. QuantumATK: an integrated platform of electronic and atomic-scale modelling tools. Journal of Physics: Condensed Matter 32, 015901 (2020).

21. Van Setten, Michiel J., Matteo Giantomassi, Eric Bousquet, Matthieu J. Verstraete, Don R. Hamann, Xavier Gonze, and G-M. Rignanese. The PseudoDojo: Training and grading a 85 element optimized norm-conserving pseudopotential table. Computer Physics Communications 226, 39-54 (2018).

22. Liechtenstein, A. Il, M. I. Katsnelson, V. P. Antropov, and V. A. Gubanov. Local spin density functional approach to the theory of exchange interactions in ferromagnetic metals and alloys. Journal of Magnetism and Magnetic Materials 67, 65-74 (1987).

23. Eriksson, Olle, Anders Bergman, Lars Bergqvist, and Johan Hellsvik. Atomistic spin dynamics: foundations and applications. Oxford university press, 2017.
